# An extraordinary new family of spiders from caves in the Pacific Northwest (Araneae, Trogloraptoridae, new family)

**DOI:** 10.3897/zookeys.215.3547

**Published:** 2012-08-17

**Authors:** Charles E. Griswold, Tracy Audisio, Joel M. Ledford

**Affiliations:** 1 Arachnology Lab, California Academy of Sciences; 2 Department of Biological Sciences, San Francisco State University; 3 Environmental Science, Policy and Management, University of California, Berkeley; 4 Summer Systematics Institute, California Academy of Sciences

**Keywords:** Haplogynae, Caves, Pacific Northwest

## Abstract

The new spider genus and species *Trogloraptor marchingtoni* Griswold, Audisio & Ledford is described as the type of the new family Trogloraptoridae. The oblique membranous division of the basal segment of the anterior lateral spinnerets of *Trogloraptor* suggests that this haplogyne family is the sister group of the other Dysderoidea (Dysderidae, Oonopidae, Orsolobidae and Segestriidae). *Trogloraptor* is known only from caves and old growth forest understory in the Klamath-Siskiyou region of Oregon and California.

## Introduction

The spider fauna of North America is a rich one, with at least 68 families including 569 genera and comprising more than 3700 species (Ubick et al. 2005). In the last generation significant progress has been made at understanding this fauna. For example, continent-wide taxonomic treatments of major families have appeared, e.g., Araneidae (Levi 1968 to present), Gnaphosidae (Platnick 1975 and subsequent) and Theridiidae (Levi 1953 and subsequent), the largest genera of jumping spiders (Salticidae) have been revised, i.e., *Habronattus* (Griswold, 1987), *Pelegrina* (Maddison, 1996) and *Phidippus* (Edwards, 2004) and the Canadian government has produced a series of guides to Canadian spiders, e.g., Dondale and Redner (1982). Perhaps most important was Vince Roth’s key to families and genera (Roth 1982), which has been updated as the comprehensive and profusely illustrated *Spiders of North America: an identification manual* (Ubick et al. 2005). This makes it relatively easy to identify to family and even to genus any spider from America north of Mexico. Nevertheless, there are still surprises in store in this well known region, especially in remote or inaccessible places, such as caves. The troglofauna of the Pacific Northwest is poorly surveyed and several arachnid groups are known to have multiple undescribed species (Ledford et al. in prep.). In this paper we describe a remarkable haplogyne spider that fits into no known family, living or extinct (Jocqué and Dippenaar-Schoeman 2006; Penney and Selden 2011). *Trogloraptor marchingtoni* gen. et sp. n., type of the new family Trogloraptoridae, is described from caves in the Pacific Northwest, and a diagnostic table (Table 1) and character discussion are presented to distinguish it from all other spider families.

**Table 1.** Diagnostic characters for Haplogynae families. *Italics represents diagnosis from Trogloraptoridae.*

Family	AME	PME distance	Cheliceral bases	Cheliceral lamina	Posterior receptaculum	3rd Entapophyses	Posterior spiracles	Posterior spiracle(s)	Emerit’s glands	Tarsal claw number	Serrula tooth rows	Labium-sternum junction	ALS basal segment	PLs AC gland spigots	CY gland spigots	Colulus
Caponiidae	present	separate	fused	present	absent	separate, short	two	advanced	absent	three	one	fused	entire	dispersed	absent	small
Diguetidae	absent	contiguous	fused	present	absent	fused	one	posterior	absent	three	one	fused	entire	dispersed	absent	small
Drymusidae	absent	contiguous	fused	present	absent	fused	one	posterior	absent	three	one	fused	entire	dispersed	absent	small
Dysderidae	absent	contiguous	free	absent	present	separate, short	two	advanced	absent	three	one	free	divided	dispersed	absent	small
Filistatidae	present	contiguous	fused	present	absent	separate, long	two	posterior	absent	three	one	fused	entire	dispersed	absent	absent
Leptonetidae	absent	contiguous	free	absent	absent	separate, long	one	posterior	present	three	one	fused	entire	in line	present	small
Ochyroceratidae	absent	contiguous	free	present	absent	separate, short	one	posterior	absent	three	one	fused	entire	in line	absent	large
Oonopidae	absent	contiguous	free	absent	present	separate, short	two	advanced	prs; abs	two	one	free	divided	dispersed	absent	small
Orsolobidae	absent	contiguous	free	absent	present	separate, short	two	advanced	absent	two	one	free	divided	dispersed	absent	small
Periegopidae	absent	contiguous	fused	present	absent	fused	one	posterior	absent	three	one	free	entire	dispersed	absent	small
Pholcidae	present	separate	fused	present	absent	absent	one	posterior	absent	three	one	fused	entire	dispersed	absent	small
Plectreuridae	present	separate	fused	present	absent	fused	one	posterior	absent	three	one	free	entire	dispersed	absent	small
Scytodidae	absent	contiguous	fused	present	absent	fused	one	posterior	absent	three	one	fused	entire	dispersed	absent	large
Segestriidae	absent	contiguous	free	absent	present	separate, short	two	advanced	absent	three	one	free	divided	dispersed	absent	small
Sicariidae	absent	contiguous	fused	present	absent	fused	one	posterior	absent	two	one; abs	fused	entire	dispersed	absent	large; abs
Telemidae	absent	contiguous	free	absent	absent	absent	two	posterior	present	three	one	fused	entire	in line	present	large
Tetrablemmidae	absent	contig./sep.	free	present	absent	fused	one	posterior	absent	three	one	free	entire	dispersed	absent	small
Trogloraptoridae	absent	separate	free	absent	absent	separate, long	one	posterior	absent	three	multiple	fused	divided	dispersed	absent	small

## Materials and methods

Species descriptions refer to a single adult individual for each sex, which is identified as a type or by the locality at which it was collected. All measurements are in millimeters and quantify the size of a structure at its widest or longest point. A section reporting the variation in the most conspicuous and variable features follows each description and represents multiple individuals (n), encompassing the full range in overall size.

Prior to examination with a Leo 1450VP Scanning Electron Microscope, all structures were cleaned with a fine brush and critical point dried. Spinneret preparations followed the methods of Griswold et al. (2005) consisting of a brief cleaning in an ultrasonicator and a gentle squeeze of the abdomen using forceps locked in place with a paperclip followed by overnight immersion in 100% ethanol in order to extend and separate the spinnerets. Large structures were examined using a Leica MZ 12.5 or MZ 16 stereomicroscope. Vulvae were carefully excised and placed in a pancreatin solution for 24–48 hours to digest extraneous tissue (Álvarez-Padilla and Hormiga 2007) then placed in water and manually cleaned. Light micrographs were prepared using a Nikon DXM1200 digital camera attached to a Leica MZ 16 stereomicroscope or a Leica DM 4000 compound microscope; multiple, stacked images were montaged using the program *Helicon Focus*®. The male pedipalp was checked for haematodochal expansion by placing it into boiling water for 2–3 minutes in a vial of hot 92% lactic acid solution (Sigma Aldrich, St Louis MO, USA), after which the pedipalp was transferred to distilled water (Ledford et al. 2011). The internal anatomy of the pedipalpal bulb and course of the reservoir were examined by immersing it in a solution of methyl salicylate (synthetic oil of wintergreen), from Mallinckrodt Baker Chemicals, Philipsburg NJ, USA (Miller et al. 2009). Abbreviations used in the text and figures are as follows: AC, aciniform gland spigots; ALE, anterior lateral eyes; ALS, anterior lateral spinnerets; AME, anterior median eyes; AT, atrium; BM, Brent McGregor; CG, Charles Griswold; ITC, inferior tarsal claw; JL, Joel Ledford; MAP, major ampullate gland spigot(s); mAP, minor ampullate gland spigot(s); Nu, nubbin (an aborted spigot); OAL, ocular area length; PER, posterior eye row; PI, piriform gland spigots; PLE, posterior lateral eyes; PLS, posterior lateral spinnerets; PME, posterior median eyes; PMS, posterior median spinnerets; RC, receptaculum; RD, Ron Davis; STC, superior tarsal claw(s). All specimens are deposited in the California Academy of Sciences (CAS).

## Phylogenetic placement

Trogloraptoridae have simple, haplogyne genitalia, with a single opening of the female vulva for fertilization and oviposition. This family differs from most other haplogyne clades. The Palpimanoidea (Archaeidae, Huttoniidae, Mecysmaucheniidae, Palpimanidae and Stenochilidae) have a foramen around the cheliceral bases, two protrusions posterior to the labral tongue, cheliceral peg teeth and a cheliceral gland mound (Wood et al. in press), all lacking in trogloraptorids. Austrochiloidea (Austrochilidae and Gradungulidae) have a clypeal hood, notched trichobothrial bases, and cylindrical gland spigots (Griswold et al. 2005), again all lacking in trogloraptorids. In spite of striking similarities in the raptorial and asymmetrical claws between gradungulids and trogloraptorids, these families are only distantly related. Trogloraptorids are not Entelegynae with secondarily simplified genitalia: the primitive tapetum in the indirect eyes (Figs 9, 11) and absence of tartipores from the spinnerets (Figs 69–74) rule this out (Griswold et al. 2005). The piriform pedipalpal bulb (Figs 9, 51–58) of the male, with tegulum and subtegulum fused, is a synapomorphy placing *Trogloraptor* in the clade Haplogynae. This clade contains, in addition to the new family Trogloraptoridae, 17 other families: Caponiidae, Diguetidae, Drymusidae, Dysderidae, Filistatidae, Leptonetidae, Ochyroceratidae, Oonopidae, Orsolobidae, Periegopidae, Pholcidae, Plectreuridae, Scytodidae, Segestriidae, Sicariidae, Telemidae and Tetrablemmidae (Platnick et al. 1991; Ramírez 2000). Haplogyne families are among the most clearly defined in the Araneae and well characterized by features of the chelicerae, spinning organs and posterior respiratory system. Trogloraptoridae are distinguished from Caponiidae, Diguetidae, Drymusidae, Filistatidae, Periegopidae, Pholcidae, Plectreuridae, Scytodidae and Sicariidae in having the chelicerae free at the base (Figs 9, 19, 23) (not fused at the base), from Dysderidae, Oonopidae, Orsolobidae, Segestriidae and Telemidae in having a single posterior spiracle (Figs 12, 60, 83) (not two posterior spiracles), from most Leptonetidae and Ochyroceratidae in having scattered AC gland spigots (Figs 73–80) (not AC gland spigots in rows on the PMS and/or PLS) and having the PME widely separated (Figs 9, 11) (not contiguous), from all Leptonetidae in having coxa-trochanter leg autospasy (not at the patella-tibia) and from Tetrablemmidae (as well as Diguetidae, Drymusidae, Periegopidae, Plectreuridae, Scytodidae and Sicariidae) in having long, separate 3^rd^ abdominal entapophyses (Figs 60, 63, 64) (not fused entapophyses). For simplicity we depict 16 diagnostic characters across the 18 families of Haplogynae in Table 1. Another striking feature is the serrula with multiple tooth rows, otherwise found in Araneomorphae only in Hypochilidae. In many ways Trogloraptoridae are a collection of primitive character states. A clue to this family’s phylogenetic affinity comes from the basal segment of the ALS: the segment is crossed diagonally by a line of membranous cuticle, contrasting with the surrounding sclerotization (Fig. 68). This morphology was first reported by Simon (1893: 310) in Dysderidae, and is a probable synapomorphy for the Dysderoidea (Dysderidae, Oonopidae, Orsolobidae and Segestriidae) (Fig. 65). It is lacking in other Haplogynae, e.g., Figures 66, 67. This peculiar spinneret morphology in Trogloraptoridae suggests that this family is allied to the Dysderoidea. If so, the family Trogloraptoridae is a primitive member of Dysderoidea, lacking the paired, anteriorly advanced posterior tracheal spiracles and posterior receptaculum of the vulva that are synapomorphies uniting the four remaining families, Dysderidae, Oonopidae, Orsolobidae and Segestriidae.

## Taxonomy

### Trogloraptoridae

Griswold, Audisio & Ledford fam. n.

urn:lsid:zoobank.org:act:0B46EFED-0BE4-4618-B14C-0EFFAA7CA4EA

#### Types.

*Trogloraptor marchingtoni* Griswold, Audisio and Ledford, here designated.

#### Diagnosis.

Ecribellate Haplogynae lacking AME, with ALE and PLE contiguous but PME separated (Figs 9, 11), chelicerae free at base and distally not forming a chela with fang (Figs 9, 10, 21, 24), Emerit’s glands absent from patellae and tibiae (Fig. 37), posterior respiratory system with broad spiracle closer to spinnerets than to epigastric furrow (Figs 12, 83), with paired, 2-branched lateral tracheal tubes and long, separate median entapophyses (Figs 60, 63), ALS basal article crossed by a diagonal membranous area (Fig. 68), and with all leg tarsi subsegmented and raptorial (Figs 13, 14, 29–32, 38, 44).

#### Synapomorphies.

The extraordinary, subsegmented raptorial leg tarsi are unique among spiders and clearly autapomorphic for the family.

### Trogloraptor

Griswold, Audisio & Ledford gen. n.

urn:lsid:zoobank.org:act:25F85266-612A-42BC-B2F9-72D3B5E0F7DC

http://species-id.net/wiki/Trogloraptor

#### Type species.

*Trogloraptor marchingtoni*new species, here designated.

#### Etymology.

The generic name refers to the cave habitat and raptorial tarsi.

#### Diagnosis.

By the characters of the family.

#### Synapomorphies.

As for the family.

#### Description.

**Figures 1–8.**
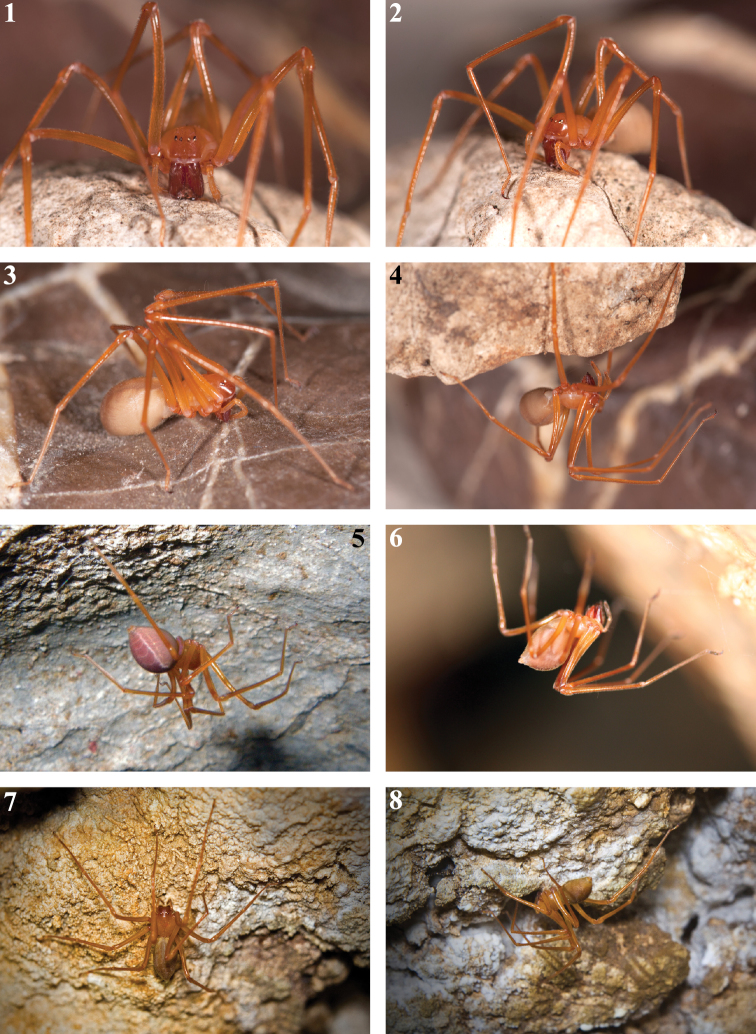
Habitus of live *Trogloraptor marchingtoni*. **1–4** female in captivity (JL) **5** female in Lake Cave (CG) **6** female in M2 Cave (RD) **7, 8** female in No Name Cave (BM).

**Figures 9–14.**
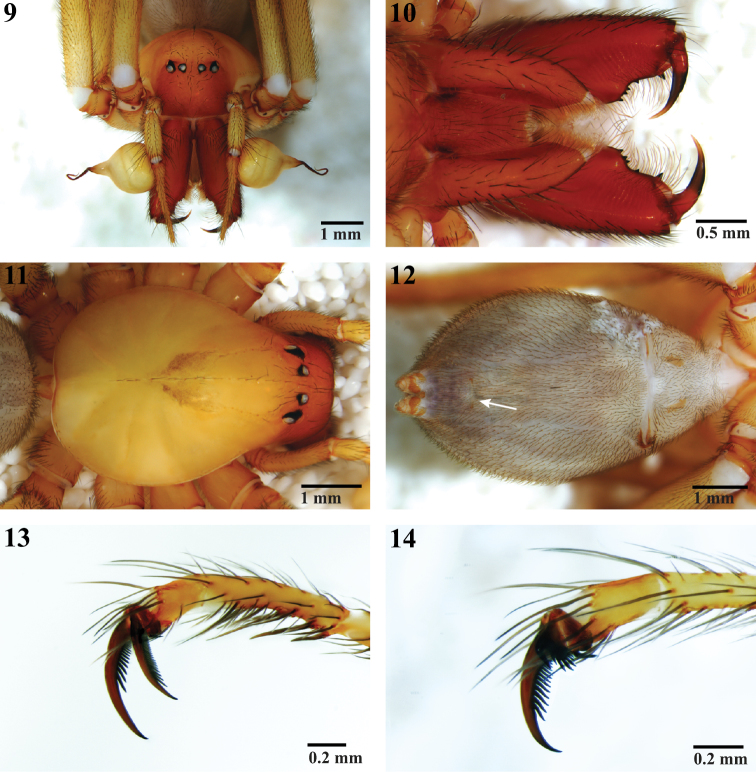
Habitus and tarsi of male *Trogloraptor marchingtoni* (CASENT9040013) from M2 Cave. **9** front **10** mouthparts, ventral view **11** carapace, dorsal view **12** abdomen, ventral view, arrow to tracheal spiracle **13** tarsus I, prolateral; and **14** tarsus IV, prolateral.

**Figures 15–18.**
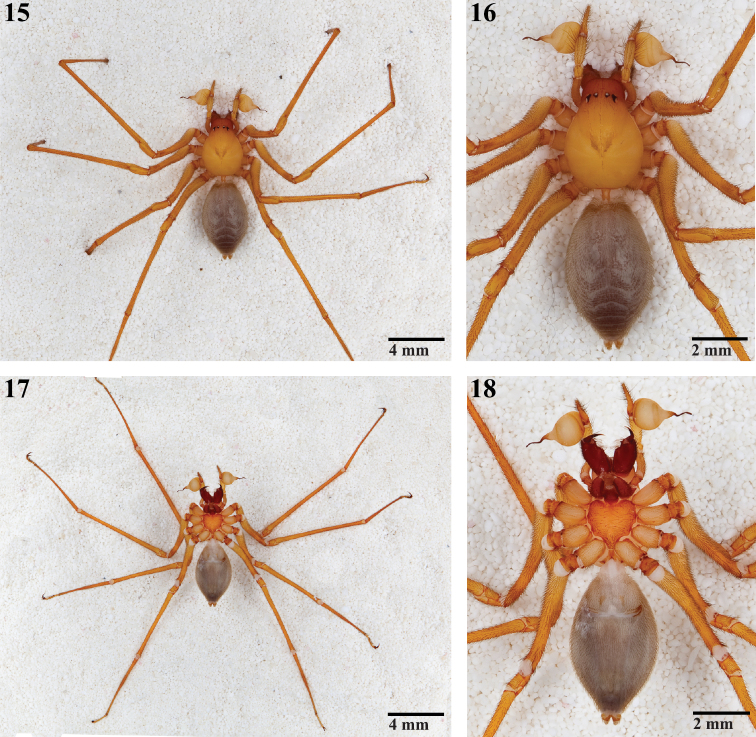
Habitus of male *Trogloraptor marchingtoni* (CASENT9040013) from M2 Cave. **15, 16 **dorsal views **17, 18** ventral views; note pedicel in Figure **16**.

Cephalothoraxwith carapace pear-shaped, narrowed anteriorly, pars cephalica faintly distinguished from pars thoracica, fovea indistinct (Figs 11, 16); six eyes, AME absent, ALE and PLE contiguous, PME separated from lateral eyes by their diameter, separated from each other by more than twice their diameter, shiny tapeta fill entire eyecup, of “primitive” type (Homann 1971) (Figs 9, 11); clypeus high, more than six times PME diameter, sloping anteriorly, ventral margin straight (Fig. 9); chelicerae free at base, without a boss (Figs 9, 22), with weak mesal lamellar ridge for basal 2/3 (Fig. 23), fang furrow with one large distal prolateral tooth and two promarginal and two retromarginal small proximal teeth; promargin with more than 30 elongate setae and setae on both margins at fang base (Figs 20, 21, 24), cheliceral gland opens as sparse pores near position of fang tip (Figs 20); fang without serrations along inner margin, apex longer than base, poison gland pore subapical, retrolateral (Figs 23, 24); no apparent chilum beneath clypeus but with small anterior sclerite between cheliceral bases, intercheliceral sclerite a narrow rectangle (Fig. 9); labrum elongate, with numerous plumose setae from base to middle, labral tongue free, longitudinally wrinkled apically, with minute, bristle-like setae distad of tongue apex (Fig. 27); pedipalpal coxa narrow, pointed apically, with membranous cuticle at apex and retroapical serrula (Fig. 10), serrula teeth in two rows (Figs 25, 26), with dorsal maxillary gland opening mesally near labrum (Figs 27, 28); labium narrow, sides converging, with weak basal notches, fused to sternum (Fig. 10); sternum heart-shaped, laterally undulate, anterior margin rounded on each side of labium, laterally with narrow lobes opposite coxae and rounded lobes between coxae, without free sclerites, posteriorly narrowly rounded between coxae IV (Fig. 18); coxae cylindrical, without retrocoxal hymen, trochanters shorter than coxae, apices straight, without notch (Fig. 18); narrow, slightly curved supracoxal sclerites above each leg coxa; leg formula 1243, legs elongate, femur I 1.69—2.30 times carapace length (Figs 1–8, 15, 17), sparsely covered with plumose setae, cuticle smooth or with fine fingerprint pattern (Figs 45–50), autospasy at coxa/trochanter joint, pairs of small sclerites visible in intersegmental membranes between coxae-trochanters and femora-patellae, metatarsi III and IV of female with ventrolateral patch of curved, spinose setae (Figs 29, 33), densest proventrally, Emerit’s glands absent from patellae (Fig. 37) and tibiae, legs with few spines except beneath tarsi I-III, pedipalp with dorsoapical spine on patella and median prolateral on tibia, female pedipalpal tarsus with three prolateral and one retrolateral spines; tarsal trichobothria absent, with only a single, subapical trichobothrium on metatarsi, 1–3 dorsal trichobothria on leg tibiae, more on pedipalpal tibia, bothrium with proximal hood (Fig. 50) or a smooth, entire ring, narrower apically (Fig. 49), trichome plumose, slightly narrowed basally (Fig. 49); tarsal organ near apex of pedipalp, at mid point of leg tarsi proximad of second membranous subsegmentation (Fig. 45), exposed, round, nearly flat, with central depressed circle or 1–2 raised sensillae (Figs 46–48); leg tarsi raptorial (Figs 5, 6, 13, 14, 29–32, 34), with flexible subsegmentations (Figs 13, 14, 30) near base and subapically in female tarsi I-IV, male I-III, subapical only in male tarsus IV, tarsi I-III with paired stout spines ventrally, one pair proximad of and three pair distad of basal subsegmentation (Figs 13, 40, 43, 44), tarsus IV lacks such spines (Figs 14, 30); leg tarsi with three claws, STC I-III slightly asymmetrical, proclaw longer (Figs 13, 43), STC teeth uniseriate, proclaws I-III with 8–9 teeth and fine basal comb, retroclaw with 15–25 fine teeth, STC IV sexually dimorphic, female claws equal (Fig. 44), male asymmetrical, retroclaw with 22 teeth, proclaw short, palmate, with one large and fan of 9 smaller teeth (Figs 14, 39), ITC long, curved, with distal and proximal teeth, tarsus without serrate accessory setae, claw tufts or scopulae (Figs 35, 36, 40–43); female pedipalp with long, smooth claw (Figs 35, 36). Abdomen oval, unsclerotized except at book lung openings, sparsely covered with setae (Figs 12, 15–18); pedicel with ventral sclerite contiguous to sternum, dorsum with lorum divided anteriorly (Fig. 16); male lacks epiandrous spigots (Fig. 82); anterior respiratory system booklungs, posterior respiratory system with broad spiracle closer to spinnerets than to epigastric furrow (Figs 12, 83), with paired, 2-branched lateral tracheal tubes and long, separate median entapophyses (Figs 60, 63), entapophyses tips frayed as muscle attachments (Fig. 64); colulus a large, oval sclerotized lobe, covered with hairs (Figs 75, 81); ALS with three segments (Fig. 69), basal segment divided obliquely by membranous cuticle (Fig. 68), with about 30 PI gland spigots, each with convex base and a narrow tapering shaft, shaft origin slightly sunken into base and encircled by a cuticular ridge (Fig. 71), female mesally with anterior large and posterior small MAP gland spigots (Figs 69, 72, 84); male resembles female except posterior MAP gland spigot replaced by a small nubbin (Fig. 70); PMS of female with two spigots with squat bases and narrow shafts (Figs 77, 78), male retains only the posterior (Fig. 80), suggesting that this is an AC gland spigot and the anterior is a mAP gland spigot, cuticle on mesal surface of PMS wrinkled (Fig. 77); PLS of female (Figs 73, 74) and male (Fig. 86) with two spigots with squat bases and narrow shafts, these probably AC gland spigots; female genitalia haplogyne, anterior edge of epigastric furrow sclerotized, vulva internally (Figs 59, 61) with median atrium and paired, lateral receptacula with sclerotized stalks and membranous apical bulbs (Fig. 62), apical bulbs may serve as muscle attachments; male pedipalp femur to tarsus lacking apophyses, cymbium narrow, without trichobothria or chemosensory scopulae, extending far beyond base of bulb (Figs 9, 15, 17, 51, 52, 57); male pedipalpal bulb piriform, swollen, lacking processes, embolus long, slender, recurved apically (Figs 51–58), spermpore subapical (Fig. 56), reservoir broad, making 1 ½ spiral within bulb (Figs 57, 58), basal haematodocha does not expand but bulb orientation twists slightly.

#### Composition.

One species described, probably another known only from juveniles.

#### Distribution.

Known only from caves and old growth forest understory in the Klamath-Siskiyou region of Oregon and California.

### Trogloraptor marchingtoni sp. n.

urn:lsid:zoobank.org:act:DD0946CA-9479-4DC5-BCA2-384B6B82599E

http://species-id.net/wiki/Trogloraptor_marchingtoni

Figures 1–64 68 [Fig F12] [Fig F13] –86

#### Types.

Holotype male from M2 Cave, 15.7 km SSW Grants Pass, Josephine Co., Oregon, USA, collected 29 July 2010 by R. S. Davis and D. S. Snyder, CASENT9040013, and paratype female from No Name Cave, Josephine Co., Oregon, 17.8 km SSW Grants Pass, collected 16 Sept. 2010 by N. Marchington, CASENT9040065, deposited in CAS.

#### Etymology.

The specific name is a patronym in honor of Neil Marchington, cave biologist, Advisory Board member of the Western Cave Conservatory, Conservation Chair, Western Region, National Speleological Society and Deschutes County Deputy Sheriff, in gratitude for his help and kindness.

#### Diagnosis.

By the characters of the genus.

**Male (Holotype).** Total length 9.70. Markings as in Figs 9–12, 15–18, cephalothorax, legs and pedipalps yellow-brown, unmarked except for dark brown v-mark posteriorly on pars cephalica, clypeus and chelicerae orange brown, abdomen purple brown with faint light chevrons posteriorly on dorsum. Carapace 4.50 long, 3.10 wide; clypeus 1.33 high; ocular area 0.45 long, 1.30 wide; ratio of eyes ALE:PME:PLE, 1.08:1.00:1.08; diameter of PME 0.18; chelicerae 2.38 long; sternum 1.75 long, 1.88 wide; labium 1.08 long, 0.70 wide; pedipalpal coxa 1.50 long, 0.45 wide; leg measurements (Femur + Patella + Tibia + Metatarsus + Tarsus = [Total]): I: 8.25 + 1.40 + 9.25 + 9.00 + 1.45 = [29.35]; II: 7.75 + 1.35 + 8.05 + 8.00 + 1.40 = [26.55]; III: 6.40 + 1.40 + 6.25 + 5.70 + 1.60 = [21.35]; IV: 7.15 + 1.40 + 6.50 + 6.35 + 1.50 = [22.90]; pedipalp: 1.55 + 0.55 + 1.70 + 2.40 = [6.20]. Pedipalp as in Figs 9, 16, 51–58.

**Variation (N=2):** Total length 6.90—9.70; carapace length 1.19--1.45 times width, height 0.33—0.35 times width; PER width 2.89—3.00 times OAL; clypeal height 6.43—7.00 times PME diameter; clypeal height 1.74—2.11 times cheliceral length; sternum length 0.92—0.93 times width, labium length 1.54—1.55 times width, pedipalpal coxa length 2.81—3.33 times width; femur I length 1.83—2.30 times carapace length; metatarsus I length 2.03—2.06 times carapace length.

**Figures 19–24.**
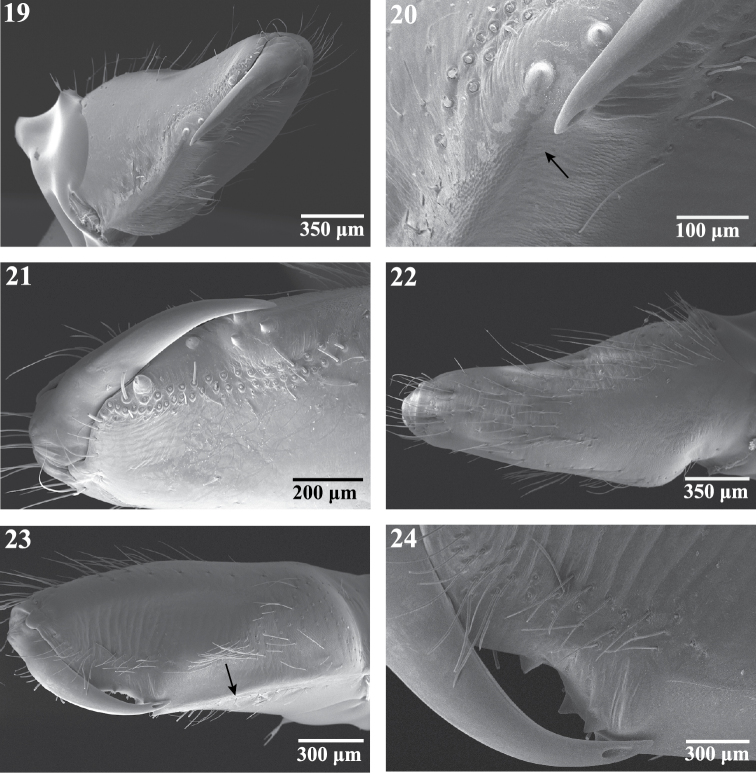
Scanning electron micrographs of the right chelicera of female *Trogloraptor marchingtoni* (CASENT9040051) from M2 Cave. **19** mesal view **20** mesal view, arrow to cheliceral gland openings **21** retrolateral view **22** ectal view **23** prolateral view, arrow to weak laminar ridge; and **24** prolateral view, close up of fang and opening of poison gland.

**Figures 25–28.**
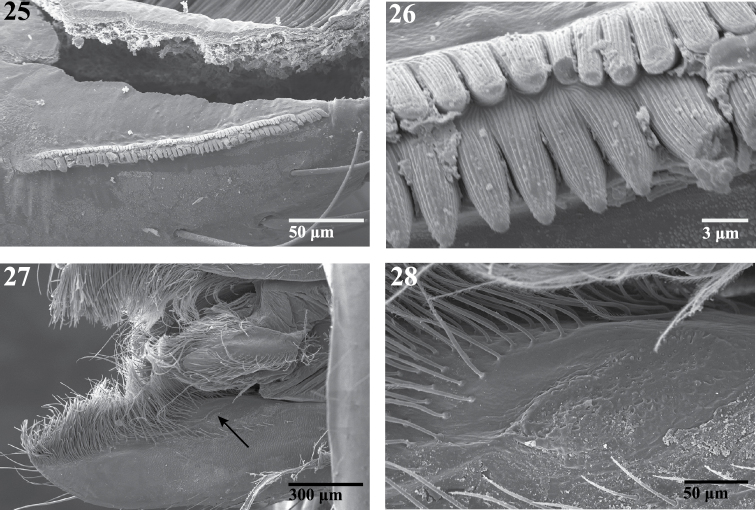
Scanning electron micrographs of the endite of a female *Trogloraptor marchingtoni* (CASENT9040051) from M2 Cave. **25** serrula **26** serrula, close up, note multiple tooth rows **27** labrum and left endite, dorsal view, arrow to maxillary gland opening **28** maxillary gland opening, close up.

**Figures 29–36.**
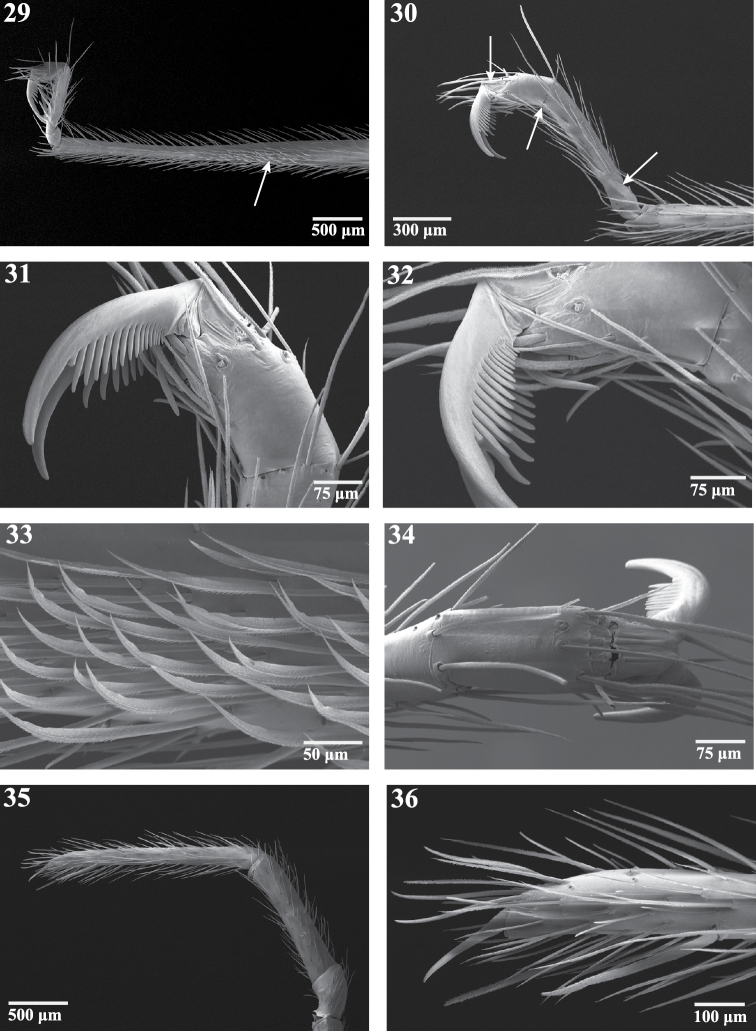
Right appendages of female *Trogloraptor marchingtoni* (CASENT9040051) from No Name Cave. **29** metatarsus and tarsus of leg III, prolateral view, arrow to ventrolateral patch of curved, spinose setae **30** tarsus of leg IV, prolateral view, arrows to membranous regions **31, 32** tarsus of leg IV, prolateral view **33** curved setae onmetatarsus of leg III, prolateral view **34** tarsus of leg IV, dorsal view **35 **pedipalp, prolateral view; and **36** tarsal claw of pedipalp.

**Figures 37–44.**
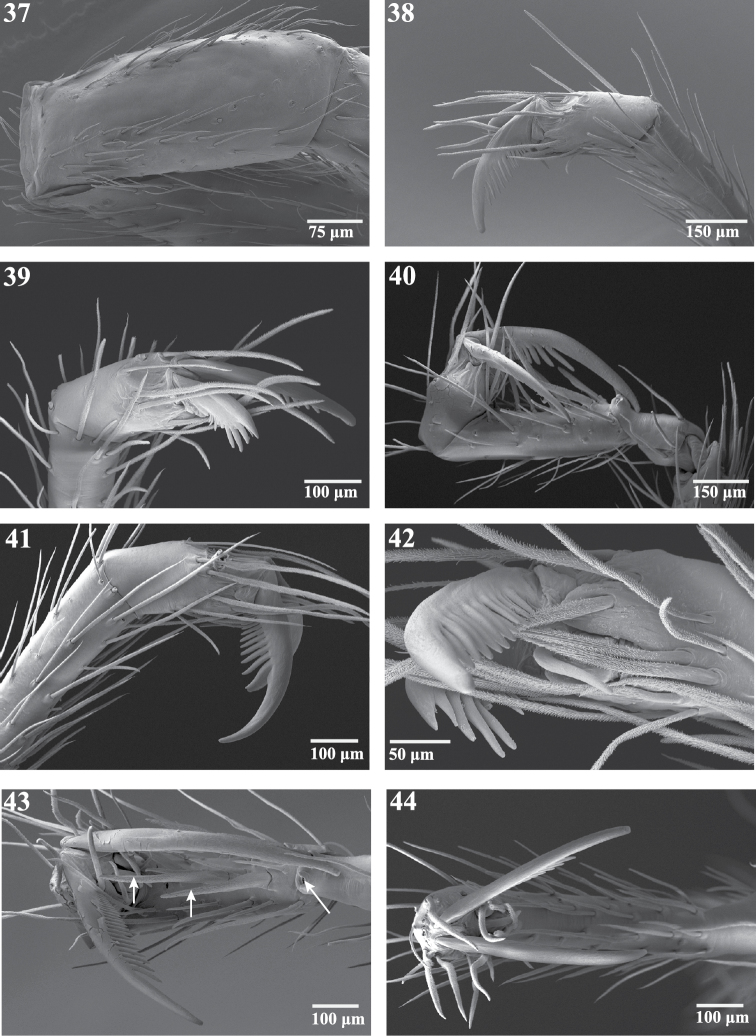
Scanning electron micrographs of legs of *Trogloraptor marchingtoni* from No Name Cave. **37** left patella IV, dorsal view **38** left tarsus IV, retrolateral view **39** left tarsus IV, prolateral view **40** right tarsus III, retrolateral view **41** right tarsus IV, retrolateral view **42** left tarsus IV, retroventral view **43** right tarsus III, ventral view, arrows to spines **44** right tarsus IV, apical view. Figures **37–39, 42** (male, CASENT9040066) **40, 41, 43, 44** (female, CASENT9040051).

**Figures 45–50.**
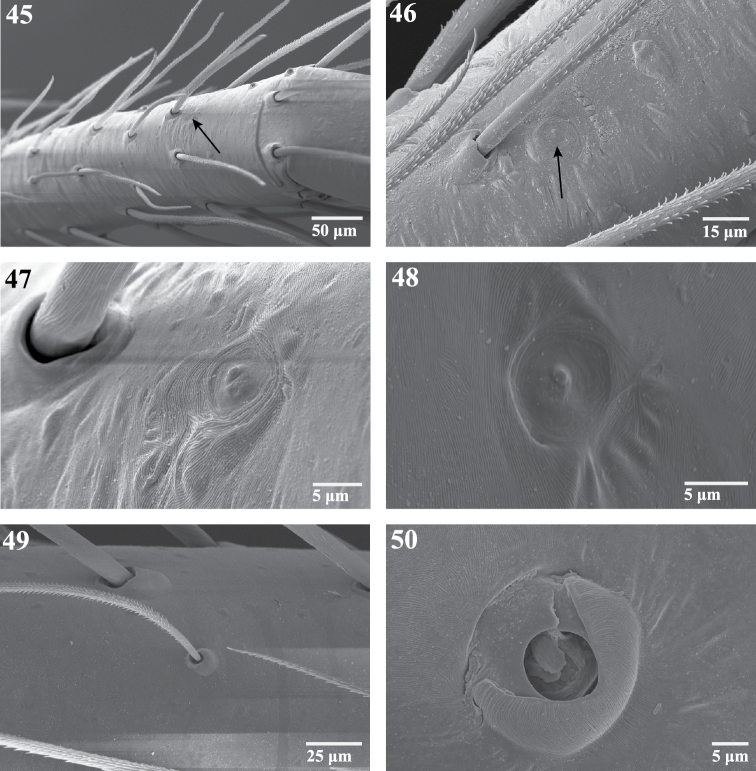
Scanning electron micrographs of sensory organs of *Trogloraptor marchingtoni*. **45** right tarsus I, arrow to tarsal organ **46** tarsus IV, arrow to tarsal organ **47** right tarsus I, tarsal organ **48** tarsal organ on palp **49** trichobothrium on tibia of R leg III, prolateral view **50** trichobothrial base on metatarsus of right leg III. Figures **45, 47–49** (female, CASENT9040051) **46** **(**male,CASENT9040066) **50** (female, CASENT9040041).

**Figures 51–58.**
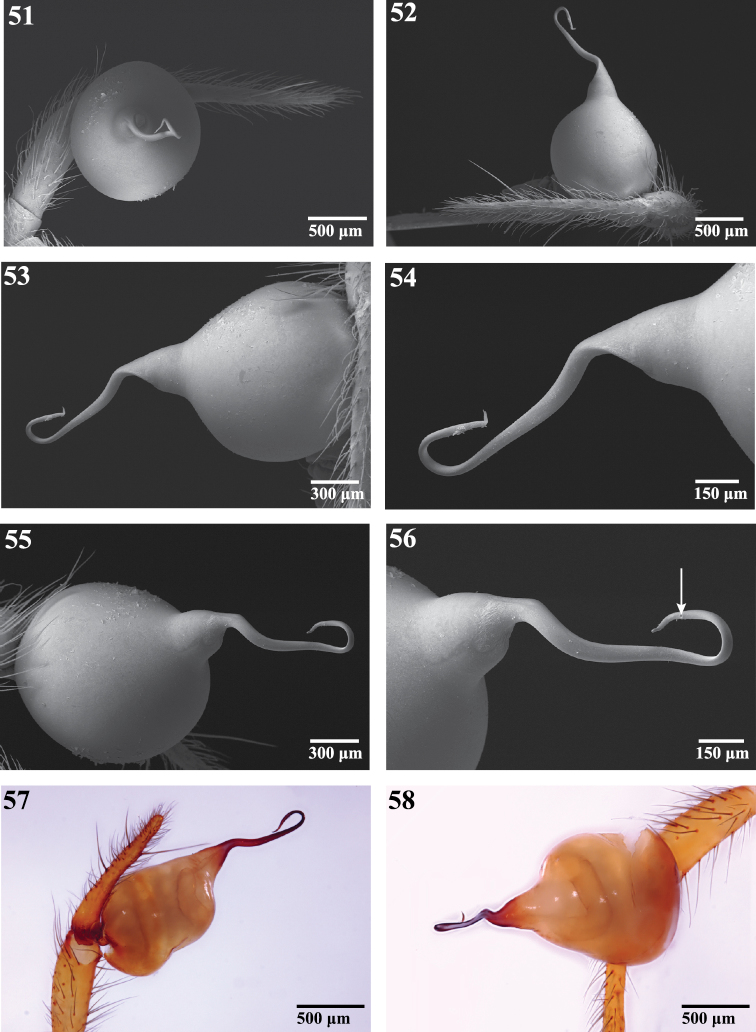
Male pedipalp of *Trogloraptor marchingtoni*: **51–56** Scanning electron micrographs of right pedipalp **51** tarsus and bulb, apical view **52** tarsus and bulb, dorsal view **53** bulb, dorsal view **54  **embolus, dorsal view **55** bulb, ventral view **56** embolus, ventral view, arrow to sperm pore **57, 58  **Automontage of left pedipalp **57** prolateral view **58** retrolateral view. Figures **51–56** CASENT9040013 from M2 Cave **57, 58** CASENT9040066 from No Name Cave.

**Figures 59–64.**
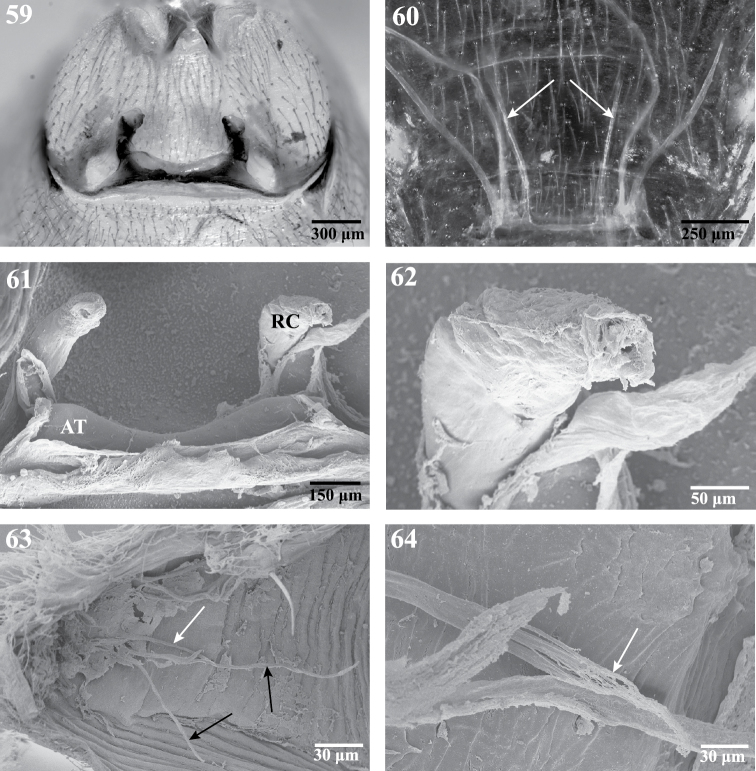
Internal anatomy of *Trogloraptor marchingtoni*, female (CASENT9040051) from No Name Cave**.** **59** vulva, dorsal view **60** female posterior respiratory system, tracheae and apodemes, with arrows to median apodemes**61–64** Scanning electron micrographs of the internal anatomy **61** vulva, dorsal view, AT, atrium, RC, receptaculum **62** apex of right receptaculum **63** posterior respiratory system, dorsal view, with white arrow to median apodeme and black arrows to lateral tracheal branches; and **64** apex of apodeme (white arrow), note frayed end typical of muscle attachment. Booklungs removed from preparation in **59, 61** and **62**.

**Figures 65–68.**
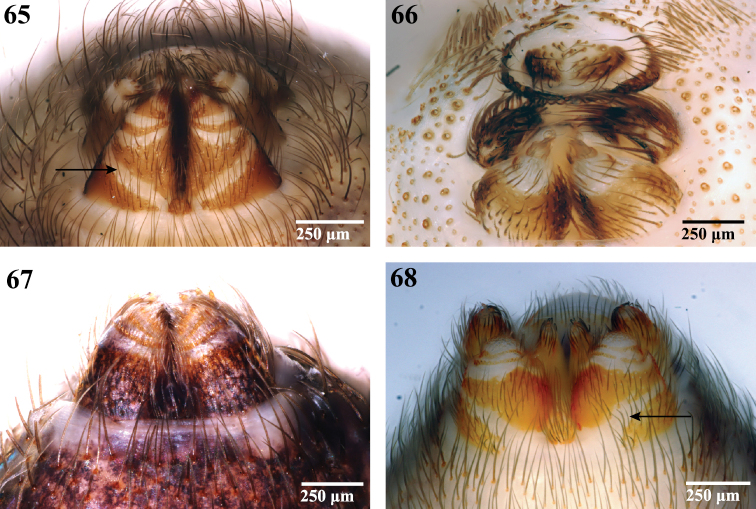
Spinnerets, ventral view. **65** Segestriidae female, *Ariadna* sp. from Tinglewood, Western Australia (CASENT 9020550) **66** Pholcidae female, *Artema atlanta* from Hawaii, USA (CASENT 9047601) **67** Drymusidae female, *Drymusa capensis* from Table Mountain, South Africa (CASENT 9043173); and **68** Trogloraptoridae male, *Trogloraptor marchingtoni* (CASENT 9040065). Arrows point to membranous band on ALS base.

**Figures 69–74. F12:**
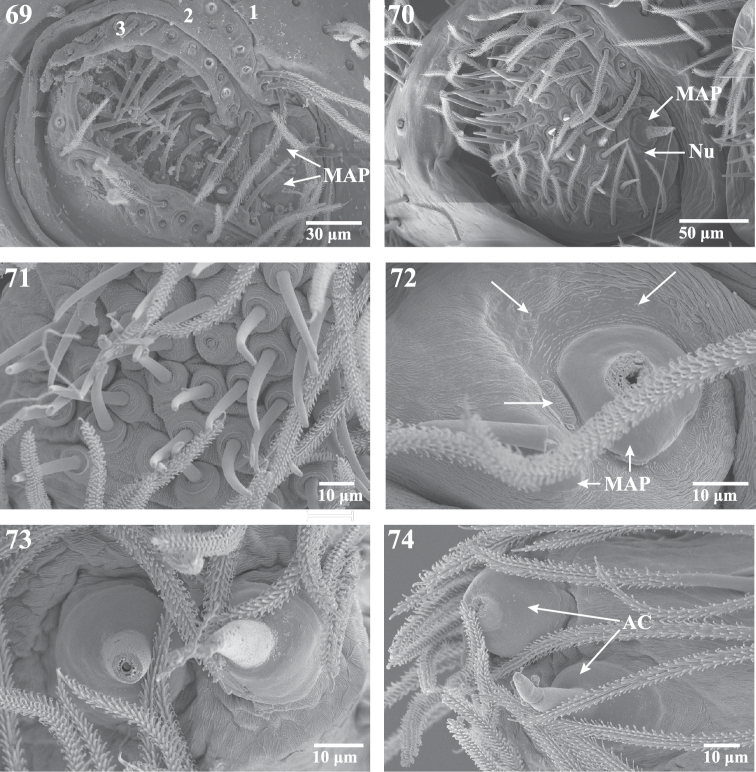
Scanning electron micrographs of the ALS and PLS of *Trogloraptor marchingtoni*, female **(**CASENT9039440) and male (CASENT9040066) from No Name Cave and penultimate female from M2 Cave (CASENT9040012). **69** penultimatefemale, right ALS, numbers refer to the three ALS segments **70** male right ALS **71** female ALS piriform gland spigots (left image flipped to appear right) **72** female major ampullate gland spigots of ALS, arrows showing individual and grouped sensillae (right image flipped to appear left) **73** female PLS apex showing aciniform gland spigots (left image flipped to appear right); and **74** female **right PLS apex**. **AC** aciniform gland spigots **MAP** major ampullate gland spigot(s) **Nu** nubbin.

**Figures 75–80. F13:**
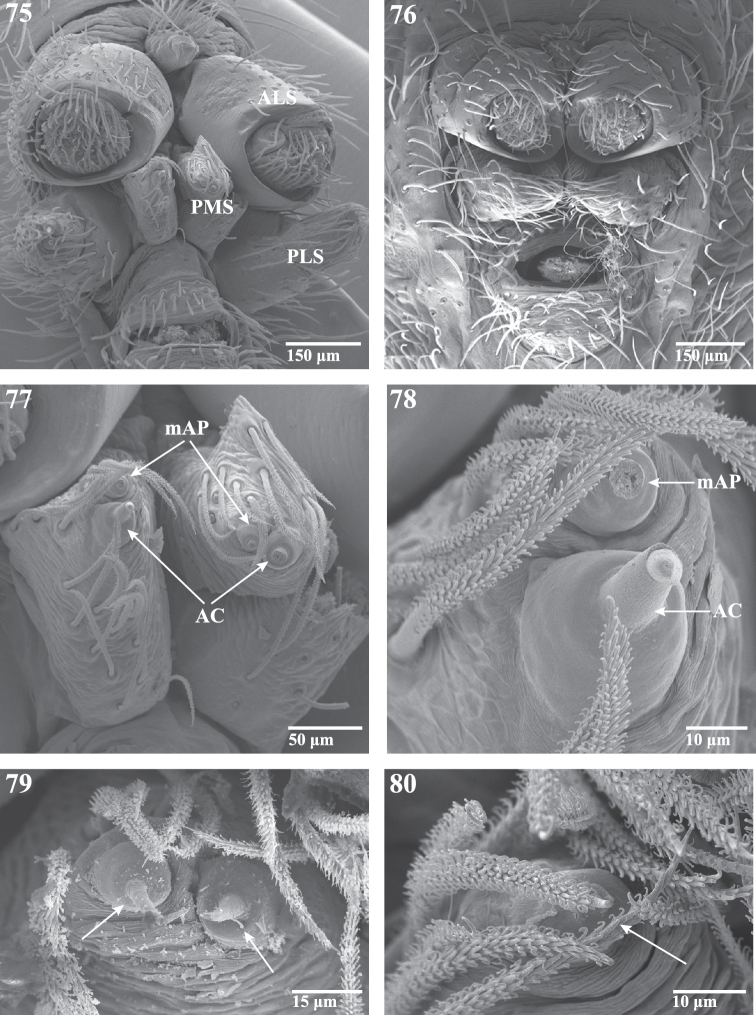
Scanning electron micrographs of spinnerets of *Trogloraptor marchingtoni* from No Name Cave, female **(**CASENT9039440), male (CASENT9040066). **75** female spinneret overview (image flipped) **76** male spinneret overview **77** female PMS overview (image flipped) **78** female left PMS (image flipped) **79** female PMS close up showing aciniform gland spigots; and **80** male left PMS apex showing single aciniform gland spigot. **AC** aciniform gland spigots **ALS** anterior lateral spinneret **mAP** minor ampullate gland spigot(s) **PMS** posterior median spinnerets **PLS** posterior lateral spinnerets.

**Figures 81–86.**
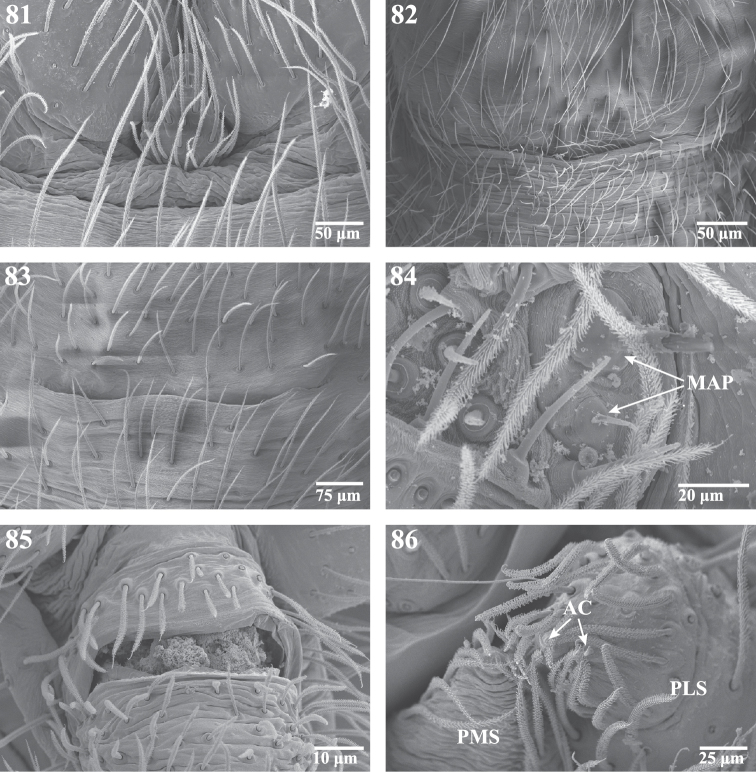
Scanning electron micrographs of the spinnerets of *Trogloraptor marchingtoni* female **(**CASENT9039440) and male (CASENT9040066) from No Name Cave, and penultimate female from M2 Cave (CASENT9040012). **81** male colulus **82** male epiandrum **83** male posterior tracheal spiracle **84** penultimatefemale right ALS **85** female anal tubercle; and **86** male left PMS and PLS spinnerets, posterior, arrows to two aciniform gland spigots on PLS. **AC** aciniform gland spigots **MAP** major ampullate gland spigot(s) **PMS** posterior median spinnerets **PLS** posterior lateral spinnerets.

#### Female

**(paratype):** Total length 9.40. Markings as in male (Figs 1–8). Carapace 3.90 long, 2.90 wide; clypeus 0.93 high; ocular area 0.38 long, 1.28 wide; ratio of eyes ALE:PME:PLE, 1.00:1.00:1.07, diameter of PME 0.15; chelicerae 2.25 long; sternum 1.75 long, 1.88 wide; labium 0.95 long, 0.65 wide; pedipalpal coxa 1.38 long, 0.50 wide. Leg measurements (Femur + Patella + Tibia + Metatarsus + Tarsus = [Total]): I: 7.15 + 1.25 + 6.85 + 7.25 + 1.35 = [23.85]; II: 6.75 + 1.30 + 6.85 + 6.95 + 1.40 = [23.25]; III: 5.25 + 1.25 + 5.10 + 4.70 + 1.15 = [17.50]; IV: 5.85 + 1.25 + 5.60 + 5.15 + 1.20 = [19.05]; pedipalp: 1.50 + 0.50 + 1.05 + 2.05 = [5.10]. Genital region weakly sclerotized externally, vulva with median translucent atrium and sclerotized lateral receptaculae, receptacular apex membranous (Figs 59, 61, 62).

**Variation (N=3):** Total length 8.27—9.60; carapace length 1.30—1.40 times width, height 0.34—0.39 times width; PER width 2.71—3.40 times OAL; clypeal height 6.17—6.91 times PME diameter, 2.05—2.43 times cheliceral length; sternum length 0.93—0.96 times width; labium length 1.28—1.54 times width; pedipalpal coxa length 2.75—3.67 times width; femur I length 1.69—1.83 times carapace length; metatarsus I length 1.72—1.78 times carapace length.

#### Natural history.

This species has been collected in the dark zone of caves, hanging beneath a few strands of silk that are attached to the cave roof (Figs 5–8). Boulders and rotting logs were searched near the entrance to M2 Cave without finding any *Trogloraptor*. Nothing has been observed of its predatory or mating behavior. Living specimens were reared in climate controlled conditions and constructed a loose tangle of web from which they hung beneath. Multiple attempts to feed the specimens a variety of prey items failed, which may indicate a preference for specific prey.

#### Distribution.

Caves in southwestern Oregon.

#### Additional material examined

(all CAS)**.** USA: OREGON: Josephine Co., No Name Cave, 17.8 km SSW Grants Pass, 16 Sept. 2010, N. Marchington, 1 ♀, CASENT9040051, 1 ♂, CASENT9040066, same data except 13 July 2011, 1♀, CASENT9039440; M2 Cave, 15.7 km SSW Grants Pass, 31 July 2010, Geo Graening, R. S. Davis and D. S. Snyder, 1 penultimate ♀, CASENT9040012, same data except 13 July 2011, N. Marchington, T. Audisio, C. Griswold, J. Ledford, D. Ubick, H. Wood and F. Álvarez-Padilla, 3 juveniles, CASENT9047599; Lake Cave near No Name Cave, 9.05 km S Wilderville, 13 July 2011, N. Marchington, T. Audisio, C. Griswold, J. Ledford, D. Ubick, H. Wood and F. Álvarez-Padilla, 1♂ (molted to maturity in captivity) CASENT9047600; Chapman Cave, 12 July 2011, N. Marchington, 2 juveniles, CASENT9039436.

#### Note.

A juvenile *Trogloraptor* specimen has been collected under debris in old growth redwood forest in far northwest California. The markings of this juvenile differ from the cave species, *Trogloraptor marchingtoni*. The northwest California specimen has dusky markings laterally on leg femora, a dusky Y marking extending back from the PLE to the posterior margin of the carapace and undulate dusky markings along the lateral carapace margin. These markings suggest that there may be at least one additional *Trogloraptor* species currently known only from the juvenile. Records for this specimen are as follows: CALIFORNIA: Del Norte Co., NE Crescent City, Jedediah Smith Redwood State Park, Ruth Perry Hatton Grove, US199 0.2 mi E junction with Walker Rd., elev. 60m, 10.29 km NNE Crescent City, old growth redwood, under woody debris, 31 March 2011, E. Garcia, C. Richart, A. Schoenhofer, D. Sitzmann, 1 juvenile, CASENT9040069 (CAS).

## Conclusions

Western North America, especially the Klamath-Siskiyou region of northern California and southern Oregon is rich in biodiversity, particularly with respect to its endemic plants and invertebrates (Myers et al. 2000). This area is particularly notable for relicts, i.e., primitive relatives of otherwise widespread taxa. The coast redwood, *Sequoia sempervirens*, is a notable example. The coastal tailed frog (*Ascapus truei* Stejneger, 1899, Ascaphidae) and mountain beaver (*Aplodontia rufa* (Rafinesque, 1817)), Aplodontiidae) are considered the world’s most primitive living frog and rodent, respectively (Nielson et al. 2001; Adkins et al. 2001). Primitive arachnids and myriapods also occur in the Pacific Northwest. Shear and Leonard (2003) described the new millipede family Microlympiidae for a minute species from the Olympic Mountains of Washington. Several families of Travunioid harvestman are found here (Briggs 1971). The pseudoscorpion genera *Pseudogarypus* (Pseudogarypidae), *Oreolpium* (Garypinidae) and *Pseudotyrannochthonius* (Pseudotyrannochthoniidae) all have relict distributions in the Pacific Northwest. These are also among the most ancient of pseudoscorpion lineages (Harvey 1998; Harvey and Štáhlavský 2009). Atypoid mygalomorphs (Coyle 2005a, 2005b; Ramirez and Chi 2004), Hypochilidae (Hedin 2001; Paquin and Hedin 2005), the 8-eyed caponiid *Calponia* (Ubick, 2005) and the cribellate leptonetid genus *Archoleptoneta* (Ledford and Griswold 2010) comprise relatively ancient spider lineages characterized by the retention of ancestral character states. If Trogloraptoridae are the most primitive living members of the Dysderoidea we have another case of a notable relict from Western North America. If such a large and bizarre spider could have gone undetected for so long, who knows what else may lurk undiscovered in this remarkable part of the world.

## Supplementary Material

XML Treatment for Trogloraptoridae

XML Treatment for Trogloraptor

XML Treatment for Trogloraptor marchingtoni
